# Primary diffuse large B-cell lymphoma of the chest wall: a case report

**DOI:** 10.1186/1477-7819-12-104

**Published:** 2014-04-22

**Authors:** Xiaoming Qiu, Yi Liu, Yanjie Qiao, Gang Chen, Tao Shi, Jun Chen, Qinghua Zhou

**Affiliations:** 1 Department of Lung Cancer Surgery, Tianjin Lung Cancer Institute, Tianjin Medical University General Hospital, No 154, Anshan Road, Heping District, 30052 Tianjin, People’s Republic of China

**Keywords:** Chest wall, Diffuse large B-cell lymphoma

## Abstract

Reports of primary diffuse large B-cell lymphomas of the chest wall are extremely rare in the literature. We report the case of a 62-year-old Chinese woman presenting with left-sided chest pain. A computed tomography scan showed a solid, round mass in the left anterior chest wall, involving the second and third costal cartilages. Complete resection and reconstruction of the chest wall was performed. The histological and immunohistochemical features of the mass were used to diagnose a primary diffuse large B-cell lymphoma.

## Background

Primary diffuse large B-cell lymphoma (DLBCL) of the chest wall is an extremely rare disease. The disease most often develops in the pleural cavity in patients with a long-standing history of pyothorax, and it is therefore thought to be pyothorax-associated [1-5]. The nonspecific clinical and radiological presentation often makes accurate diagnosis difficult; excision or incisional biopsies are needed for a definite pathologic diagnosis. Patients have a relatively good prognosis, especially when the diagnosis is made at a local stage suitable for surgical resection. We report the case of a patient with a rare primary DLBCL of the chest wall and describe her treatment course.

## Case presentation

**Figure 1:**
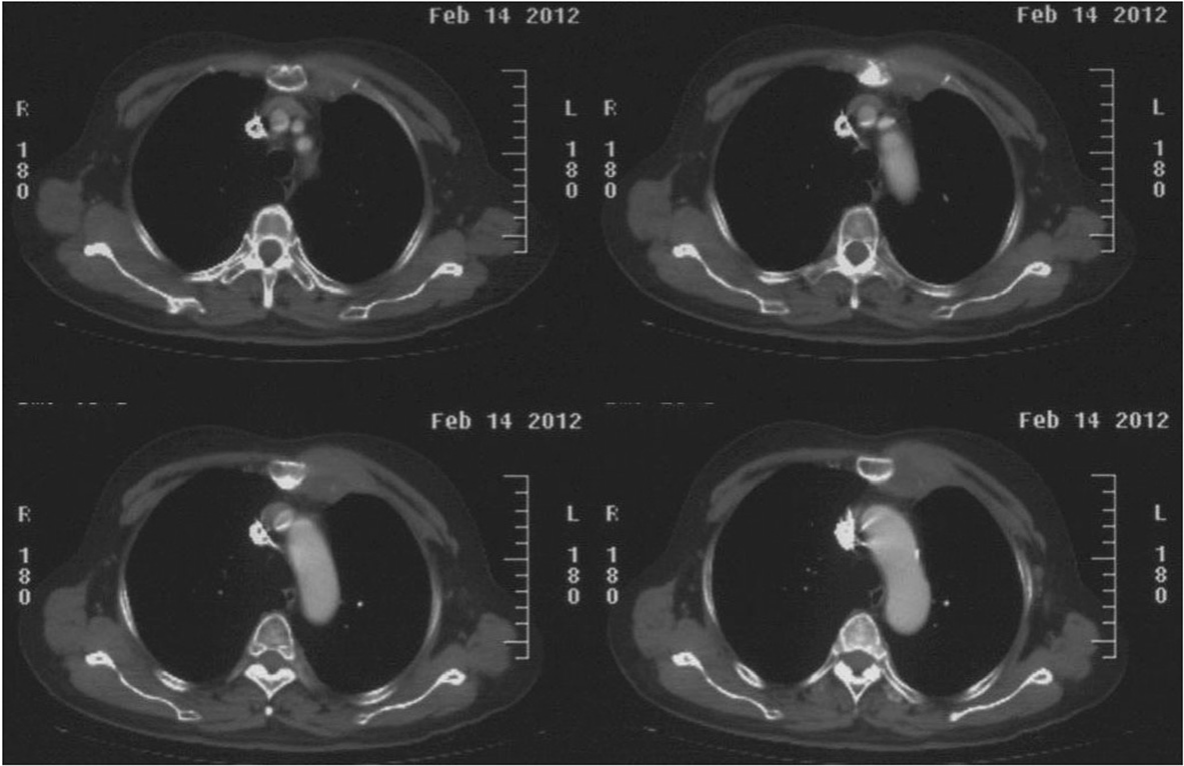
Computed tomography scan showing a solid, round mass in the left anterior chest wall.

A 62-year-old Chinese woman presented to our department complaining of intermittent left-sided chest pain for the past six months. No other symptoms such as fever, cough, dyspnea, or weight loss were present. Her medical history showed five years of well-controlled hypertension. She had no personal history of trauma or surgery, and she had no family history of cancer. Her physical examination revealed a palpable, immobile, rubbery, subcutaneous mass in the left anterior chest wall, measuring approximately 7 cm × 7 cm. An evaluation with a computed tomography (CT) scan revealed a solid, round mass in the left anterior chest wall, involving the second and third costal cartilages. Some bone destruction was noted and considered to be a sign of malignancy (Figure 1). Her abdominal and brain CT scan were negative for metastasis.

We decided on surgical intervention for both diagnosis and treatment; a standard median sternotomy was performed on 20 February 2012. The tumor was located in the left anterior chest wall, involving the second and third costal cartilages, with the medial border of the tumor reaching the sternum and costal cartilage junction. The tumor was resected en-bloc with the surrounding tissues. A reconstruction of the chest wall was performed using polyethylene terephthalate surgical mesh.

**Figure 2:**
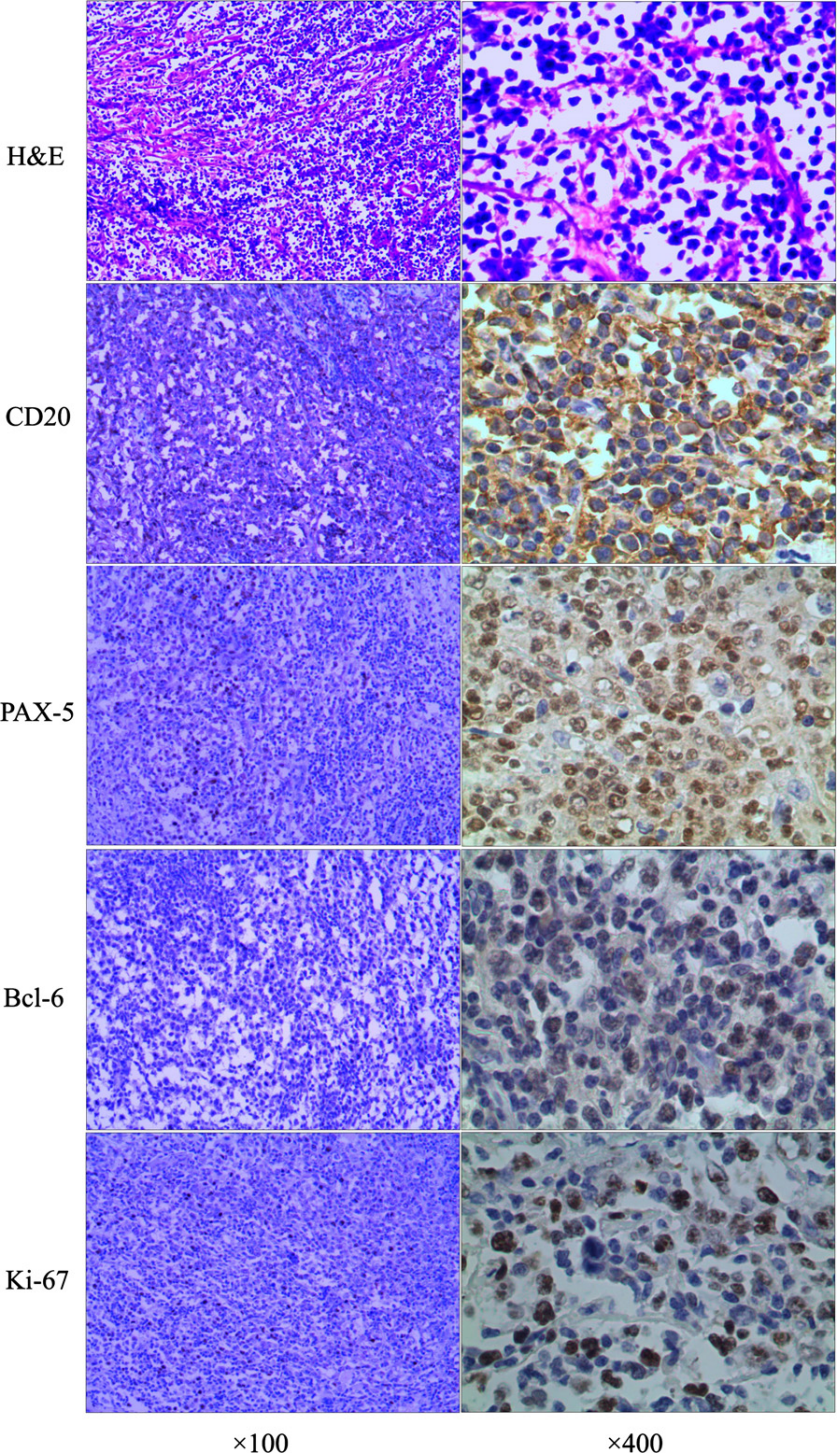
**Hematoxylin and eosin (H&E) staining showing a highly pleomorphic large-cell proliferation.** Immunohistochemistry was diffusely positive for CD20, paired box protein 5 (PAX-5), and B-cell lymphoma 6 protein (Bcl-6). The Ki-67 index was between 60 and 70%. (Magnification shown at ×100 and ×400).

The gross tumor measured 75 mm × 70 mm × 15 mm, with pleura covering the posterior surface. The cut surface was soft and yellowish-gray in color, with visible areas of bone tissue representing the resected ribs. A pathological examination revealed a highly pleomorphic large-cell proliferation. Immunohistochemistry was diffusely positive for CD20, paired box protein 5 (PAX-5), and B-cell lymphoma 6 protein (Bcl-6) (Figure 2). The Ki-67 index was between 60 and 70%. The tumor cells were negative for a cluster of differentiation 3 (CD3), CD10, CD117, CD43, CD68, myeloperoxidase (MPO), lysozyme, multiple myeloma oncogene 1 (MUM-1), and CD138. From these findings, we diagnosed the tumor as DLBCL with an immunohistological staining pattern consistent with germinal center B-cell derivation.

Her postoperative course was uneventful. Adjuvant chemotherapy was administered after surgery when she was referred to the Department of Hematology. At 17 months after surgery, there is no evidence of local recurrence or distal metastasis.

## Discussion

Primary lymphoma of the chest wall is quite rare. In a patient series described by Press *et al.*, 4 of 250 patients (1.6%) with lymphoma only had the disease in the chest wall; this included a single patient with non-Hodgkin lymphoma [6]. In another retrospective report, 7 of 157 patients with non-Hodgkin lymphoma initially presented with a large chest-wall mass. In these few reports of primary lymphoma of the chest wall, DLBCL is the most common subtype [7].

DLBCL is a group of large, lymphoid B-cell malignant proliferations that is clinically, morphologically, and genetically heterogenous. It constitutes about 30% of all non-Hodgkin lymphomas and is the most common histologic subtype [8]. Most reported DLBCLs of the chest wall are pyothorax-associated lymphomas (PALs) - tumors that develop in the pleural cavity of patients with long-term pyothorax. This pyothorax, in turn, results from artificial pneumothorax created for the treatment of lung tuberculosis or tuberculous pleuritis. PAL is strongly associated with the latency III form of the Epstein-Barr virus (EBV) infection [1-5,9,10]. Cytokines such as interleukin 6 (IL-6) and IL-10, produced at the site of chronic inflammation, may induce a local immunosuppressive environment that plays an important role in lymphomagenesis [11-13]. An elevated serum neuron-specific enolase level in patients with chronic pyothorax may be an indicator of PAL development [14]. Most PALs have an immunohistological staining pattern consistent with late germinal center and/or post germinal center B-cell derivation [15-17].

Fujimoto *et al.*[18] reported the case of a patient with EBV-associated DLBCL that developed in the chest wall after a polyethylene terephthalate surgical mesh was implanted during surgery for a squamous cell lung carcinoma. In that case, the patient’s disease resembled a typical PAL in many aspects such as the location, immunostaining pattern, and the latent III form of EBV infection. The authors concluded that surgical implants can be a cause of localized, long-standing inflammation, which might enable EBV-transformed B-cells to escape from host immune surveillance. This could then lead to lymphomagenesis, as seen in PAL. Fouad *et al.*[19] reported the case of another patient with DLBCL that developed in the chest wall. The patient had a history of blunt trauma with swelling and rib fracture, 41 years prior. The immunophenotype was similar to PAL - a non-germinal center phenotype. The authors proposed that post-traumatic chronic inflammation lead to tumor proliferation, with a similar mechanism to that seen in PAL.

Our patient is unusual when compared to the previously reported instances of chest-wall DLBCL. She has no history of pyothorax or chest-wall trauma that could be a cause of chronic inflammation. Unlike typical PAL, the immunohistological staining pattern in our patient indicates a germinal center B-cell derivation.

The malignancy’s stage and histopathological diagnosis have a major impact on its treatment and prognosis. Patients with lymphoma are usually treated with chemotherapy or local irradiation. It remains controversial whether patients with lymphoma located only in the chest wall should receive surgical resection. Hodgson *et al.*[20] reported the outcomes in 324 patients with clinical stage I-II Hodgkin lymphoma who were treated with chemotherapy and local irradiation. They found that patients with chest-wall invasion had poor local control and survival. Romagurea *et al.*[21] reported that surgical debulking is associated with improved survival in stage I-II diffuse large-cell lymphoma.

In the few reported cases of DLBCL of the chest wall, surgery provided a relatively satisfactory outcome. Luh *et al.*[1] reported a patient with DLBCL developing from a long-standing pyothorax of the left lower chest wall. In that case, the patient remained free of local recurrence or metastasis at nine months after surgical resection, without further chemotherapy [5]. In Hsu *et al.*’s series [7], 3 of the 4 patients with isolated chest wall lymphoma were managed with surgical resection and adjuvant chemotherapy. No tumor recurrence was recorded during the follow up period, which had a maximum duration of 171 months. Treatment after surgical resection is often based on a combination chemotherapy regimen. DLBCL is frequently treated using CHOP (cyclophosphamide, doxorubicin, vincristine, and prednisone); rituximab may improve the response to CHOP treatment, as has been shown in systemic DLBCL.

Our patient’s staging included an abdominal and brain CT scan, which gave no indication of metastasis. She underwent en-bloc resection of the tumor and chest wall with adjuvant chemotherapy. She remains without evidence of local recurrence or distal metastasis, at more than one year after treatment. We conclude that surgery followed by adjuvant chemotherapy can provide a satisfactory outcome in patients with DLBCL isolated to the chest wall.

## Conclusions

When complete resection can be achieved, surgery should be the treatment of choice in patients with localized DLBCL. To the best of our knowledge, primary DLBCL of the chest wall is extremely rare, with few cases reported in the literature. Our patient is unusual, both in clinical presentation and immunophenotype.

## Consent

The patient granted written informed consent for publication of this manuscript and the accompanying images. A copy of the written consent is available for review by the Editor-in-Chief of this journal.

## Abbreviations

Bcl-6: B-cell lymphoma 6 protein; CHOP: cyclophosphamide, doxorubicin, vincristine, and prednisone; CT: computed tomography; DLBCL: Primary diffuse large B-cell lymphoma; EBV: Epstein-Barr virus; H&E staining: Hematoxylin and eosin staining; IL-6: interleukin 6; MPO: myeloperoxidase; MUM-1: lysozyme, multiple myeloma oncogene 1; PALs: pyothorax-associated lymphomas; PAX-5: paired box protein 5.

## Competing interests

The authors declare that they have no competing interests.

## Authors’ contributions

XQ and YL collected all data and authored the manuscript. YQ and GC were responsible for patient care and analysis of follow-up data. TS provided histopathologic confirmation. JC and QZ performed the surgical procedure, also contributing to data analysis and shaping of the manuscript. All authors have read and approved the final manuscript.
